# Acoustic voice analysis of women with migraine

**DOI:** 10.1055/s-0046-1827041

**Published:** 2026-08-31

**Authors:** Helena Cysneiros, Taciana Maciel, Pedro Souza, Pedro Sampaio Rocha-Filho, Marcelo Moraes Valença, Daniella Araújo de Oliveira

**Affiliations:** 1 Universidade Federal de Pernambuco, Centro de Ciências Médicas, Programa de Pós-Graduação em Neuropsiquiatria e Ciências do Comportamento, Recife PE, Brazil.; 2 Universidade Federal de Pernambuco, Centro de Ciências da Saúde, Programa de Pós-Graduação em Fisioterapia, Recife PE, Brazil.; 3 Universidade Federal de Pernambuco, Centro de Informática, Programa de Pós-Graduação em Ciência da Computação, Recife PE, Brazil.

**Keywords:** Headache Disorders, Primary, Migraine Disorders, Voice, Phonation

## Abstract

**Background:**

Migraine and voice are related because of the shared pathophysiological mechanisms involving the vagus nerve, which also innervates the larynx.

**Objective:**

To conduct acoustic voice analysis and evaluate voice-related quality of life in women with migraine, in comparison with a control group.

**Methods:**

In the current cross-sectional study, we performed voice recordings and used the Voice-Related Quality of Life and migraine characteristics questionnaires. The variables included loudness, fundamental frequency, jitter, shimmer, glottal-to-noise excitation ratio, and phonatory deviation diagram.

**Results:**

The sample comprised 193 women (55 in the control group and 138 in the migraine group). No statistically significant differences were observed between the groups regarding the acoustic variables studied. The migraine group reported poorer scores in the physical domain of the Voice-Related Quality of Life questionnaire ( *p* = 0.003) compared to the control group. The Random Forest statistical model predicted group allocation with 76% of precision.

**Conclusion:**

In the studied sample, no differences were observed between women with migraine and the control group regarding the parameters of acoustic voice analysis. Women with migraine presented poorer voice-related quality of life compared to the control group.

## INTRODUCTION

Voice is a complex and multifaceted phenomenon that is objectively assessed using acoustic analysis. This assessment provides valuable information about many conditions, and it may complement neurological disease diagnoses, such as those of Parkinson's and Alzheimer's diseases. 1 Voice assessments are used to analyze several relevant aspects, such as patient-reported outcome measures, including quality of life, and acoustic parameters, such as fundamental frequency (f0), loudness, and noise-related parameters (such as jitter and shimmer).

The investigation of voice (phonation and speech) in headache disorders, particularly migraine, is a promising and emerging field. 2 Migraine is a neurological disorder characterized by neuronal events that include the dilation of cerebral blood vessels, which leads to pain. 3 Migraine episodes usually last from 4 to 72 hours and are typically unilateral, worsened by routine physical activity, mild to severe in intensity, and accompanied by nausea, vomiting, and increased sensitivity to light, sound, or odors. 4 The disease deeply impacts daily-life activities and affects over 1 billion people worldwide, mostly women. 5

The literature suggests that individuals with migraine often present specific behavioral patterns. A high frequency of episodes, for example is associated with more neglect of physical discomfort that may trigger another episode, reinforcing the migraine cycle. 6 These behaviors corroborate the theory that migraine involves altered interoceptive processing (that is, perception of internal bodily sensations). 7 8 Interoception depends on descending predictions of bodily states and ascending visceral signals, which are modulated by the vagus nerve. 9

Understanding the role of the vagus nerve and the trigeminocervical complex in migraine pathophysiology is important to comprehend the relevance of studying voice in migraine. The trigeminal nerve contributes to migraine-related nociception, 10 which is associated with hyperexcitability of the trigeminocervical complex. 11 Anatomically, the trigeminal and vagus nerves communicate at the nucleus of the solitary tract and the spinal trigeminal nucleus; both innervate the dura mater. 12

The vagus nerve innervates many vital organs and transmits sensory information from the viscera to the central nervous system. Systemic autonomic symptoms commonly associated with migraine (nausea, vomiting, abdominal pain, gastroparesis, and diarrhea) are regulated by vagal activity. 13 This nerve is also responsible for the full innervation of the larynx, which is fundamental for voice production. 14 15

Migraine is associated with dysfunction of the autonomic nervous system (ANS), mediated by the vagus nerve. This dysfunction may result from vasovagal alterations triggered by migraine or by migraine-related pain. Furthermore, pain, which is often reported in individuals with migraine, may disrupt ANS regulation, 13 contributing to the complex and poorly-understood relationship between migraine and voice.

The quantification of neurological disorders using acoustic voice analysis is a rapidly-growing research field with potential applications in large-scale health monitoring. 2 As precision medicine becomes increasingly important for migraine diagnosis and intervention, objective and scalable diagnostic tools must be researched and developed. 16 In this context, acoustic voice analysis may be useful for screening and diagnostic assistance, clinical evaluation and monitoring of migraine, enabling better patient management.

Therefore, the current study aimed to conduct acoustic voice analysis and evaluate voice-related quality of life (V-RQOL) in women with migraine, in comparison with a control group; the hypothesis is that migraine influences vocal function.

## METHODS

The present quantitative, cross-sectional study was conducted in the city of Recife, Brazil, from May 2023 to November 2024, and it was approved by the Ethics in Research Committee of Universidade Federal de Pernambuco (under no. 7.017.767). The study followed the Declaration of Helsinki and the Strengthening the Reporting of Observational Studies in Epidemiology (STROBE) statement, and all participants provided signed informed consent.

### Participants

The study involved adult women aged 18 to 52 years who were not undergoing menopause, as self-reported. Menopause was an exclusion criterion because hormonal alterations result in vocal changes. The participants were divided into migraine group (MG) and control group (CG). Individuals in the MG were recruited from a headache outpatient clinic. They were diagnosed with migraine, with or without associated tension-type headache, by a neurologist following the International Classification of Headache Disorders, version 3. 4 The CG included family members or employees of the same clinic without migraine characteristics and with a history of episodic, non-migraine headaches.

The exclusion criteria were history of voice disorders or prior voice therapy or neurological, musculoskeletal, or cognitive conditions that could impair the understanding or execution of data-collection procedures. Pregnant women and those with biological children under 1 year of age were also excluded due to the potential vocal changes caused by hormonal fluctuations during pregnancy and the postpartum period. 17 Participants who were menstruating or had upper-airway conditions on the day of the assessment were rescheduled. Individuals experiencing an acute migraine episode during data collection were reassessed once symptom-free to minimize discomfort and reduce bias. The sample size was defined by convenience.

### Instruments

Data collection included sociodemographic and vocal-health information, as well as the following instruments:

#### *Voice-related quality of life (V-RQOL)*

The V-RQOL questionnaire assesses the impact of voice on quality of life, and it has been validated in Brazil. 18 19 It includes two domains: socioemotional and physical. The overall score ranges from 0 to 100 points, and high scores indicate better quality of life. The scores are categorized as *group A* (71–100 points; good quality of life), *group B* (36–70 points; moderate quality of life), or *group C* (0–35 points; poor quality of life). 20 The Cronbach's alpha for internal consistency was greater than 0.9 for all domains. 19

#### *Migraine characteristics questionnaire*

The research team developed a structured questionnaire based on International Headache Society 4 criteria to gather information on the clinical features of migraine in the MG.

### Procedures

#### *Acoustic voice analysis*

Voice recordings were obtained in a quiet environment using a Lenovo computer, a Karsect HT-9 headset microphone positioned 5 cm from the mouth at a 45-degree angle to the sagittal plane of the head, and an Andrea Pure Audio noise reduction device. The recordings were made using VoxMetria 5.4 (CTS Informática), with a sampling rate of 44 kHz and 16-bit resolution. The participants were instructed to inhale and sustain the vowel /ε/ without varying pitch or intensity, for as long as they felt comfortable. They also counted from 1 to 10 to provide a connected-speech sample.

**Figure 1:**
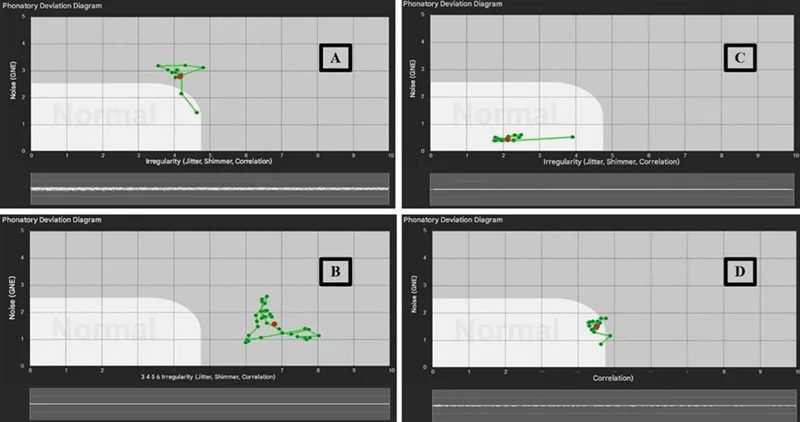
Phonatory-deviation diagrams (PDDs) of the participants in the migraine group (MG, **A,B** )– and the control group (CG; **C,D** ). The MG presented altered PDDs, while the CG presented normal PDDs.

The study variables included:

- Fundamental frequency (f0): Measured in Hertz (Hz), f0 represents the number of vocal-fold vibrations per second. 21 The normal range for adult female voices is between 150 and 250 Hz; 22
- Jitter: Represents short-term variability in f0, expressed as a percentage. Values up to 0.6% are considered normal; 23
- Shimmer: Expressed as a percentage, it indicates amplitude variation between vocal-fold vibration cycles. Normal values are up to 6.5%. 22 23 Both jitter and shimmer are glottal signal-disturbance measures, and they correspond to the disturbances occurring cycle by cycle;
- Glottal-to-noise excitation ratio (GNE): This measure of vocal noise indicates the similarity between sound-signal cycles, representing the ratio of glottal signal energy to noise energy. 24 The GNE is measured in dB, with values ≥ 0.5 dB considered normal; 23 and
- Phonatory deviation diagram (PDD): The PDD is a two-dimensional graphical representation of acoustic data that correlates four acoustic measures. The x-axis represents jitter, shimmer, and their correlations, while the y-axis represents GNE values. 22 Figure 1 shows examples of PDDs. Normal voices appear on the lower left quadrant (light gray).

To ensure data accuracy, the first and last 2 seconds of each voice emission were excluded, as they may reflect irregularities related to vocal attack or residual air. For the analysis, at least 5 seconds of each sample were considered.

### Data analysis

Data analysis was performed using descriptive and percentage-based statistics; statistical significance was set at 5%, with 95%CIs. The Shapiro-Wilk test assessed data normality, and the Chi-squared (χ ^2^ ) tests were applied to the categorical variables. Data were organized using Microsoft Excel (2019; Microsoft Corp.), and the statistical analyses were performed using IBM SPSS Statistics for Windows (IBM Corp.) software, version 20.0.

Machine-learning algorithms were used to assess whether the data could support predictive modeling. Moreover, logistic regression was used to model relationships between independent variables and a binary dependent variable. 25 Decision-tree models, including Random Forest, were used to capture complex, non-linear relationships by combining multiple decision trees to enhance accuracy and generalizability

Due to an imbalance in the number of participants in the CG and MG, the Synthetic Minority Oversampling Technique (SMOTE) algorithm was used to balance the groups by generating synthetic observations based on existing data. 26

Accuracy and F1-score metrics were applied to assess model performance. The F1-score (that is, the harmonic mean of precision and recall) assesses classification balance when both measures are equally important. Accuracy quantifies the overall precision of a classification model, calculated as the number of correct predictions divided by the total number of predictions. 27

## RESULTS

**Figure 2:**
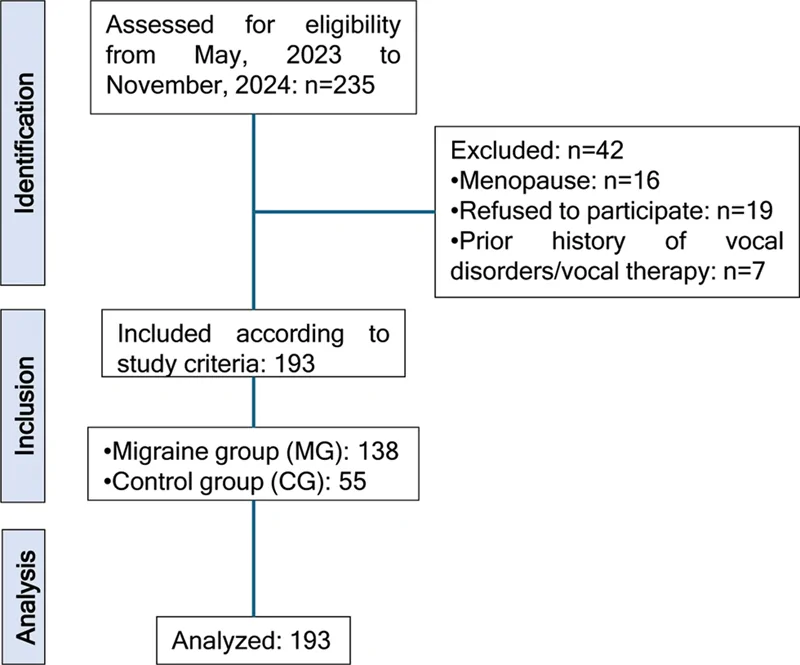
Participant-selection flowchart.

Overall, 193 participants underwent acoustic voice analysis (mean age of 32 ± 9.5 years). The mean age was of 31 ± 9.1 years in the MG, and of 32 ± 9.4 years in the CG. A participant-selection flowchart according to the STROBE guidelines is provided in Figure 2 .

Table 1 represents the sample's sociodemographic characteristics, including income, occupation, and years of schooling. In Brazil, income is typically reported as monthly earnings rather than hourly wages, a convention maintained in the characterization of the sample.

**Table 1:** Sociodemographic characterization of the sample (n = 193)

Average household income (R$)	CG (n = 55)		MG (n = 138)		Total (n = 193)	
	n	%	n	%	n	%
≤ 1,320 (monthly minimum salary in 2023, Brazil)	9	16.4	24	17.4	33	17.1
1,321–2,550	17	30.9	28	20.3	45	23.3
2,551–5,000	16	29	49	35.5	65	33.7
≥ 5,001	13	23.6	37	26.8	50	25.9
**Occupation**	**CG (n = 55)**		**MG (n = 138)**		**Total (n = 193)**	
	**n**	**%**	**n**	**%**	**n**	**%**
Student	12	22	26	19	31	38
Healthcare professional	16	29	42	30	35	58
Retired/Housewife	5	9	9	7	5	14
Domestic worker	1	2	4	3	5	5
Other	21	38	57	41	28	78
**Years of schooling**	**CG (n = 55)**		**MG (n = 138)**		**Total (n = 193)**	
	**n**	**%**	**n**	**%**	**n**	**%**
≤ 9	0	0	6	4.3	6	3.1
10–12	9	16.4	37	26.8	46	23.8
13–18	31	56.4	51	37	82	42.5
≥ 19	15	27.3	43	31.2	58	30
Not reported	0	0	1	0.7	1	0.5

Abbreviations: CG, control group; MG, migraine group; R$, Brazilian Reais.

Table 2 shows the migraine characteristics of the MG. Among the 138 participants, 98 received a diagnosis of migraine, 25, of chronic migraine, and 11, of probable migraine. Information on the type of migraine was missing for four participants.

**Table 2:** Data from the migraine characteristics questionnaire answered by the migraine group (n = 138)

Migraine characteristics	n	%
Intensity		
Mild	10	7.2
Moderate	92	66.7
Severe	32	23.2
Not reported	4	3.0
**Duration (hours)**		
≤ 3	33	24.0
4–72	74	53.6
≥ 73	27	19.6
Not reported	4	3.0
**Laterality**		
Unilateral	80	58.0
Bilateral	54	39.0
Not reported	4	3.0
**Type of pain**		
Pulsatile	89	64.5
Tightness/Pressure	45	32.6
Not reported	4	3.0
**Location**		
Frontal	39	28.3
Temporal	5	3.6
Occipital	11	8.0
Holocranial	21	15.2
Frontoparietal	5	3.6
Fronto-occipital	11	8.0
Parietal	25	18.1
Parieto-occipital	8	5.8
Frontotemporal	5	3.6
Not reported	8	6.0

Table 3 presents the results of the acoustic voice analysis. Alterations in f0 and GNE were less frequent in both groups, and the MG demonstrated a higher percentage of alterations in jitter, shimmer, and PDD values relative to the controls. These findings may be related to the disproportionate group sizes, as the migraine sample was nearly 2.5 times larger, and it is plausible to assume that a greater number of alterations might be observed in a larger sample. No statistically significant differences were found between the groups.

**Table 3:** Alteration frequency and mean values of acoustic vocal analysis parameters divided by group (n = 193)

Vocal parameter	CG (n = 55)			MG (n = 138)			*p*
	n	%	Mean (±)	n	%	Mean (±)	
Loudness	53	96.3	38.4 ± 5.2	133	96.4	38.1 ± 5.4	1.0
F0	3	5.4	113.5 (43.1)	3	2.2	60.1 (1.79)	0.2
Jitter	9	16.3	0.55 (0.9)	36	26.1	0.7 (1.5)	0.1
Shimmer	10	18.2	5.1 (4.5)	32	23.2	5.4 (5.6)	0.4
Glottal-to-noise excitation ratio	2	3.6	0.8 (0.1)	9	6.5	0.8 (0.1)	0.4
Phonatory deviation diagram	12	21.9	Not applicable	44	31.8	Not applicable	0.2

Jitter alterations were present in 16.3% of the CG and in 26.1% of the MG, while shimmer alterations were observed in 18.2% of the CG and in 23.2% of the MG. For the GNE, alterations were present in 3.6% of the CG and in 6.5% of the MG. The PDD presented alterations in 31.8% of the MG and in 21.9% of the CG.

The Random-Forest model predicted the presence of migraine based on acoustic voice parameters (loudness, f0 jitter, shimmer, GNE, and PDD) with 76% of precision. The F1-scores for the Logistic-Regression, Decision-Tree, and Random-Forest models were of 0.56, 0.47, and 0.74 respectively. The corresponding accuracy scores were of 0.64, 0.54, and 0.76 respectively. Regarding the V-RQOL questionnaire ( Table 4 ), the MG showed poorer scores in the physical domain compared to the CG ( *p* = 0.003).

**Table 4:** Data from the Voice-Related Quality of Life (V-RQOL) questionnaire

		Control group (n = 55)		Migraine group (n = 138)		Total (n = 193)	
Domain	Category	n	%	n	%	n	%
Overall	Good quality of life	50	90.9	113	81.9	163	84.5
	Moderate quality of life	5	9.1	24	17.4	29	15.0
	Poor quality of life	0	0.0	1	0.7	1	0.5
Socioemotional	Good quality of life	55	100.0	128	92.8	183	94.8
	Moderate quality of life	0	0.0	9	6.5	9	4.7
	Poor quality of life	0	0.0	1	0.7	1	0.5
Physical*	Good quality of life	53	96.4	104	75.4	157	81.3
	Moderate quality of life	2	3.6	30	21.7	32	16.6
	Poor quality of life	0	0.0	4	2.9	4	2.1

Note: *Chi-squared test; *p* = 0.003.

## DISCUSSION

The current study aimed to conduct acoustic voice analysis and evaluate V-RQOL in women with migraine, in comparison with a control group. There were no differences in the parameters of acoustic voice analysis between the groups. In contrast, the MG demonstrated poorer V-RQOL compared with the CG.

Although visual laryngeal assessments were not performed and structural alterations cannot be entirely ruled out, it is reasonable to speculate that the participants did not present vocal alterations. This assumption is supported by the absence of self-reported voice problems (according to the exclusion criteria), as well as a minimal f0 variation observed in both groups (n = 3 per group, all below the normal range).

Alterations in f0 are associated with several vocal disorders. 28 This parameter is primarily determined by the size, mass, and tension of the vocal folds and by subglottic pressure, 21 resulting from complex interactions involving laryngeal muscle activity and vocal-fold vibration. Increased stiffness of the vocal-fold mucosa or subglottic pressure raises the f0, while enlarged mass or length lowers it, 29 contributing to the auditory perception of a low-pitched voice. In the present study, only the acoustic vocal analysis was performed.

Jitter alterations may reflect variations in vocal-fold mass or tension and impaired neuromuscular control of the larynx. 29 For shimmer, which quantifies cycle-to-cycle amplitude variation and is associated with irregularities in glottal vibration, elevated values may indicate increased noise in the acoustic signal resulting from irregular vocal fold vibration. 22 23

For the GNE, a parameter that assesses the degree to which the vocal signal originates from vocal-fold vibration versus turbulent airflow in the vocal tract, alterations provide insight into the overall regularity of the sound wave. 24

The PDD is commonly used in clinical practice to provide a more objective description of vocal quality, monitor therapeutic progress, and quantify vocal deviation. It also enables the comparison of a vocal sample with normative reference areas and helps identify different phonatory mechanisms and degrees of dysphonia. 30 The PDD incorporates the GNE, which indicates vocal noise, jitter, shimmer, and their correlation, which reflect phonatory instability. Therefore, PDD alterations suggest phonatory instability, possibly due to neuromuscular imbalances involving the larynx and vocal tract.

The findings observed in part of the MG are suggestive of vocal alterations, which may be related to vocal risk behaviors or to maladaptive compensatory mechanisms secondary to a preexisting condition, such as migraine. 22 31 This level of vocal alteration may occur occasionally or be almost imperceptible, and it is possible to perform routine vocal activities with minimal difficulty, rare fatigue, and without interruptions. 22

Individuals living with chronic pain-related conditions, similar to migraine, such as fibromyalgia, chronic fatigue syndrome, irritable-bowel syndrome, are more likely to develop functional voice disorders like muscle-tension dysphonia and less likely to present structural laryngeal lesions. 32

In addition to autonomic dysfunction, individuals with migraine frequently report stress and anxiety, which are also associated with cervical-muscle tension. 33 This tension contributes to laryngeal musculoskeletal imbalance, which may lead to vocal dysfunction or dysphonia, particularly of the functional and organofunctional types. 34 Dysphonia associated with migraine is an underrecognized clinical presentation that has rarely been reported in the literature. 35 The limited existing evidence lacks clinical descriptions and does not clarify the specific manifestations of dysphonia.

The Random-Forest model identified individuals in the MG with 76% of precision, which is in line with previous literature 36 and supports the hypothesis that migraine affects vocal production. Since none of the recordings were collected during a migraine episode, these findings suggest that vocal alterations may occur even outside of episodes.

The MG showed poorer scores in the physical domain of the V-RQOL questionnaire compared to the CG. These results may reflect a heightened awareness of physical sensations among participants, as such sensations tend to be more objective and easier to perceive and recall. In contrast, the socioemotional domain is subjective and harder to assess, 20 facilitating the overlooking of related symptoms. These findings reinforce the relevance of vocal assessment in individuals with migraine. Acoustic voice analysis serves as a valuable tool for healthcare professionals in disease evaluation and monitoring. 37

There is a consensus among headache specialists that a multidisciplinary approach offers advantages and leads to better outcomes, because it facilitates the prevention of headache-related complications and enables the promotion of the patient's overall health. 38 From this perspective, to the best of our knowledge, the present study was the first to include a speech-language pathologist with specialization in voice as a member of the research team in a study on primary headaches. It was also the first to demonstrate that acoustic voice analysis may be able to distinguish the voices of individuals with migraine from those without it, with 76% precision.

Although research on the vocal manifestations of migraine is very limited and carries theoretical and clinical implications, the present study offers preliminary evidence that may support further investigation of voice as a potential biomarker for migraine.

Considering the highly-disabling and impactful nature of migraine, 5 we speculate that these preliminary findings may provide a basis for future research aimed at developing voice-analysis tools and applications for clinical screening or patient self-monitoring throughout treatment. Such tools would be useful to assist in the diagnosis and monitoring of migraine through vocal samples, taking into consideration the fact that acoustic voice assessment is a quick, simple, and low-cost method, which facilitates its incorporation into the clinical routine.

### Study limitations

A limitation of the current study is that the participants did not undergo visual laryngeal assessment (such as, videolaryngoscopy or nasofibrolaryngoscopy), which may have restricted the identification of undiagnosed vocal-fold pathologies. In addition, the sample size was limited, particularly in the CG. Moreover, the V-RQOL questionnaire was developed to be administered to dysphonic individuals. In the current research, it was used on subjects with no vocal complaints and no history of voice disorders or voice therapy. Additionally, the menstrual cycle influences vocal quality beyond the menstruation phase alone. Each participant was assessed only once, which hampered an accurate tracking of their menstrual cycle, possibly introducing a confounding bias. Due to the cross-sectional design, the findings obtained through the Random-Forest model are exploratory, and causal relationships involving migraine and vocal alterations cannot be confirmed.

### SUGGESTIONS FOR FUTURE RESEARCH

Future studies should adopt longitudinal designs and incorporate laryngeal examinations capable of detecting structural or functional abnormalities, or employ validated dysphonia-screening protocols or similar methods, and/or perceptual-auditory analysis across all phases of a migraine episode. These studies should use more comprehensive voice diagnostic tools and improved control of confounding biases, such as vocal-fold lesions, stress, anxiety, medication use (prophylaxis or not), and water and coffee intake, as they may affect the degree of vocal-fold hydration. Considering that pain may influence voice quality, applying a headache diary, either physical or digital, is encouraged, to control for the frequency of episodes.

In conclusion, in the sample studied, the parameters of acoustic voice analysis did not differ between the MG and CG. However, the MG showed poorer V-RQOL compared to the CG. Furthermore, a Random-Forest model achieved 76% of precision in predicting migraine based on the assessed vocal parameters.

Considering its cross-sectional design, the current study was not able to establish a causal relationship between migraine and vocal alterations. These findings may help the development of complementary diagnostic methods and new strategies to monitor the disease in the future, based on acoustic voice analysis.
